# Interim analysis of the multinational, post-authorization safety study (NISSO) to assess the long-term safety of sonidegib in patients with locally advanced basal cell carcinoma

**DOI:** 10.1186/s12885-024-13101-z

**Published:** 2024-11-14

**Authors:** Ralf Gutzmer, Ulrike Leiter, Peter Mohr, Katharina C. Kähler, Paolo Antonio Ascierto, Massimiliano Scalvenzi, Ketty Peris, Gemma María Pérez-Pastor, Ricardo Fernández-de-Misa, Rafael Botella-Estrada, Robert E. Hunger, Serena Martelli, Nur Güneli, Ramon Arntz, Axel Hauschild

**Affiliations:** 1 Universitätsklinik für Dermatologie, Venerologie, Allergologie und Phlebologie, Johannes Wesling Klinikum, Minden, Germany; 2 Universitäts-Hautklinik Tübingen, Tübingen, Germany; 3 Elbe Klinikum, Buxtehude, Germany; 4 Department of Dermatology, University of Kiel, Kiel, Germany; 5 Unit of Melanoma Cancer Immunotherapy and Development Therapeutics, Istituto Nazionale Tumori IRCCS Fondazione G. Pascale, Naples, Italy; 6 Department of Dermatology, University of Naples Federico II, Naples, Italy; 7 Dermatologia, Dipartimento Di Medicina E Chirurgia Traslazionale, Università Cattolica del Sacro Cuore, Rome, Italy; 8 Dermatologia, Dipartimento Di Scienze Mediche E Chirurgiche, Fondazione Policlinico Universitario A. Gemelli-IRCCS, Rome, Italy; 9 Servicio de Dermatologia, Hospital General Universitario de Valencia, Valencia, Spain; 10 Hospital Universitario Nuestra Señora de Candelaria, Santa Cruz de Tenerife, Spain; 11 Hospital Universitario La Fe Dermatología, Valencia, Spain; 12 Department of Dermatology, Inselspital, Bern University Hospital, University of Bern, Bern, Switzerland; 13 Sun Pharmaceutical Industries B.V, Hoofddorp, Netherlands; 14 SUN Pharmaceuticals Germany GmbH, Leverkusen, Germany

**Keywords:** Basal cell carcinoma, Hedgehog pathway inhibitors, Sonidegib

## Abstract

**Background:**

Following the pivotal phase II trial BOLT, the Hedgehog (Hh) inhibitor sonidegib was approved in the EU to treat locally advanced basal cell carcinoma (laBCC) in patients not amenable to surgery or radiotherapy. We report safety data from the interim analysis of the real-world NISSO study.

**Methods:**

NISSO is an ongoing non-interventional, multinational, post-authorization safety study (NCT04066504). Patients with laBCC are treated with sonidegib 200 mg orally once daily and followed for 3 years. Dose modifications were allowed according to the local prescribing information.

**Results:**

Between May 6, 2019, and March 15, 2022, 321 patients with laBCC were enrolled at 46 European sites (data cut-off: June 22, 2023). Treatment was discontinued in 241 (75.1%) patients, with the main reasons being the patient/guardian decision (n = 69, 28.6%), treatment success (n = 40, 16.6%) and the physician decision (n = 35, 14.5%). The median duration of sonidegib exposure was 8.8 months (4.4–13.7 months). Overall, 284 (88.5%) patients had ≥ one treatment-emergent adverse event (TEAE). Most TEAEs were ≤ grade 2 and the most common were muscle spasms (n = 141; 43.9%), dysgeusia (n = 119; 37.1%), and alopecia (n = 97; 30.2%). After 3 months of treatment, the cumulative rates of muscle spasms, dysgeusia, and alopecia were 21.8%, 16.2%, and 3.7%, respectively. TEAEs led to treatment discontinuation in 59 (18.4%) patients, while 149 (46.4%) patients had at least one TEAE leading to dose reduction or interruption. Serious drug-related TEAEs were reported in 13 (4.1%) patients.

**Conclusions:**

These results confirm the safety profile previously observed. Most patients experienced the onset of common TEAEs after 3 months of treatment, and the cumulative incidence of most common TEAEs was 10–20% lower compared to the BOLT study, except for dysgeusia and fatigue that had a similar incidence. The percentage of patients experiencing TEAEs requiring interruption or dose reduction was similar to the BOLT study, while the proportion of patients with TEAE leading to discontinuation of sonidegib was lower. This study demonstrates that the tolerability of sonidegib is manageable in routine clinical practice.

Trial registration.

NCT04066504.

## Background

Skin cancers are the most common malignancy in Europe, Australia and North America. Non-melanoma skin cancers (NMSC) represent the vast majority of them, with basal cell carcinomas (BCC) accounting for more than 80%, and squamous cell carcinomas (SCC) for up to 20% [1]. It is estimated that one in three Caucasians will develop BCC in their lifetime [2]. BCC frequently afflicts patients with Gorlin syndrome (also called nevoid basal cell carcinoma syndrome [NBCCS]), a rare autosomal dominant condition caused by inactivating mutations in the Patched (*PTCH*) gene (incidence: 1/57,000 to 1/256,000) [3]. Loss of function of *PTCH* results in uncontrolled Hedgehog (Hh) signal transduction, which is linked to the development of BCC. Almost all BCCs (both NBCCS and sporadic BCCs) are dependent on Hh signalling for growth and survival [2].

Surgical excision and/or radiotherapy form the mainstay of standard primary treatment of BCCs, yielding cure rates of > 95% and up to 90%, respectively [4]. Occasionally, BCC progress into such an advanced stage that curative surgery and radiotherapy are no longer feasible. Advanced BCC includes locally advanced BCC (laBCC) and metastatic BCC (mBCC). Sonidegib and vismodegib are specific inhibitors of an oncogenic protein named Smoothened, which is involved in the Hh signaling pathway. Both drugs are approved by the US Food and Drug Administration (FDA) and the European Medicines Agency (EMA) for the treatment of patients with laBCC who are not eligible for surgery or radiotherapy. Vismodegib is also approved for mBCC, whereas sonidegib is approved for mBCC only in Switzerland and Australia. According to the latest European BCC guidelines [5], Hh inhibitors (HHIs) are recommended in patients not amenable to surgery or radiotherapy and who are classified by the European Association of Dermato-Oncology (EADO) as stage II (nodular BCC in critical areas of the head, poorly defined margins, recurrent lesions, aggressive histotypes, multiple syndromic or sporadic lesions, perineural invasion) and EADO stage III (laBCC). Additionally, vismodegib is recommended in EADO stage IV patients (mBCC) [5].

The approval of sonidegib was based on the phase II, multicentre, double-blind, and multiple cohort clinical trial BOLT conducted in patients with laBCC or mBCC [6]. Using ERIVANCE-like criteria, the objective response rate in patients with laBCC receiving sonidegib 200 mg once daily was 60.6 (95% confidence interval [CI]: 47.8–72.4) by central review and 74.2 (95% CI: 62.0–84.2) by investigator review [7]. Based upon the 42-month analysis of the registration study BOLT, a total of 79 adult patients were exposed to sonidegib for a median of 11 months. The 42-month safety results demonstrated that sonidegib is associated with an acceptable and manageable safety profile in the intended target population characterized by predictable, primarily events of low to moderate grade, which are generally reversible [7]. However, safety data from patients with long-term exposure to sonidegib are limited. Here, we report the interim analysis of the NISSO long-term post-authorization safety study (PASS) in order to further characterize the long-term safety and tolerability profile of sonidegib under real-world (routine clinical practice) conditions.

## Methods

NISSO is an ongoing non-interventional, multinational, post-authorization safety study (NCT04066504). Eligible patients were aged 18 years or older with a diagnosis of laBCC and who were not amenable to curative surgery or radiation therapy. Patients were treated with sonidegib 200 mg orally taken once daily. Dose modifications according to the approved local country prescribing information were permitted. Sonidegib treatment was started either at the first visit for this study or prior to study entry. Patients with Gorlin syndrome could be enrolled if all other criteria were met. Patients treated with any HHI besides sonidegib within 3 months prior to study entry were excluded. Patients were followed up for the duration of 3 years after enrolment. The evaluable safety population includes all patients who received at least one dose of sonidegib during the study. The primary objective is to assess the long-term safety and tolerability profile of sonidegib in the treatment of laBCC as determined by the occurrence of adverse events (AEs), serious AEs, deaths and discontinuation. The study protocol was approved by the institutional review boards or independent ethics committees of participating study centres and the study was undertaken in accordance with the provisions of the Declaration of Helsinki and Good Clinical Practice guidelines. All patients provided written informed consent.

## Results

Between May 6, 2019, and March 15, 2022, 321 patients with laBCC were enrolled and treated with sonidegib at 46 study sites in Germany, Italy, Spain, and Switzerland (data cut-off: June 22, 2023). Table 1 shows baseline demographics and characteristics.

**Table 1:** Baseline demographics and characteristics

	*N* = *321*
Age, years, median (range)	77 (33–101)
Gender, n (%)	
Male	198 (61.7)
Female	123 (38.3)
Gorlin syndrome, n (%)	39 (12.2)
Primary tumour localization, n (%)	
Head and neck	233 (72.5)
Trunk and abdomen	30 (9.3)
Multiple locations	22 (6.8)
Extremities	19 (5.9)
Genital region	4 (1.2)
Unknown	13 (4.1)
BCC histotype (multiple answers possible), n (%)	
Nodular	79 (24.6)
Infiltrative	78 (24.3)
Superficial	26 (8.1)
Basosquamous	16 (5.0)
Morphoeic	15 (4.7)
Micronodular	9 (2.8)
Multifocal	8 (2.5)
Multiple histotypes	3 (0.9)
Unknown	92 (28.7)
Other	36 (11.2)
Largest diameter of primary tumour, mm, median (range)	22.0 (0.25–400.0)
Prior surgery, n (%)	130 (40.5)
Prior systemic therapies, n (%)	53 (16.5)
Sonidegib	6 (1.9)
Vismodegib	41 (12.8)
Immunotherapy	6 (1.9)
Cemiplimab	4 (1.2)
Pembrolizumab	2 (0.6)
Prior radiotherapy, n (%)	32 (10.0)
Prior other local therapy, n (%)	30 (9.3)
Other prior therapies*, n (%)	4 (1.2)

^*^Acitretin, imiquimod, photodynamic therapy, topical sonidegib

Median age in the study population was 77 years with 61.7% of male patients and 12.2% affected by Gorlin syndrome. Prior to sonidegib, 40.5%, 16.5% and 10.0% of patients received surgery, systemic therapy and radiotherapy, respectively. The median duration of sonidegib treatment was 8.8 months (interquartile range [IQR]: 4.4–13.7 months) including days off treatment and 7.2 months (IQR: 4.2–12.8 months) excluding days off treatment. Median time on study (time from start of sonidegib treatment until either date of last contact for patients who ended the study or date of last visit for patients remaining in study) was 18.9 months (IQR: 12.3–27.9 months). At the time of data cut-off, treatment was ended in 241 (75.1%) patients, among which the reasons were patient/guardian decision (n = 69; 28.6%), treatment success (n = 40; 16.6%), physician decision (n = 35 14.5%), disease progression (n = 30; 12.5%), toxicity (n = 22; 9.1%), lost to follow-up (n = 19; 7.9%), death (n = 13; 5.4%; deemed not drug-related by investigators), regular end of study (3 years of follow-up after enrolment) (n = 8; 3.3%), organizational reason (n = 3; 1.2%), and missing reason (n = 2; 0.8%). Overall, 284 (88.5%) patients had ≥ one treatment-emergent adverse event (TEAE) (Table 2).

**Table 2:** Overview of TEAE

	*N* = 321
	*n (%)*
Patients with TEAE	284 (88.5)
Patients with drug-related TEAE	252 (78.5)
Patients with TEAE leading to death*	17 (5.3)
Patients with TEAE leading to discontinuation of sonidegib^‡^	59 (18.4)
Patients with TEAE leading to dose reduction	73 (22.7)
Patients with TEAE leading to interruption	98 (30.5)
Patients with serious TEAE	87 (27.1)
Patients with serious drug-related TEAE^#^	13 (4.1)

^*^Considered not drug-related by investigator

^‡^The only TEAE leading to discontinuation that occurred in more than 2% of patients was basal cell carcinoma (*n* = 17, 5.3%)

^#^Myocardial infarction, vertigo, nausea, vomiting, fatigue, alanine aminotransferase increased, aspartate aminotransferase increased, blood creatine phosphokinase increased, hepatic enzyme increased, muscle spasms, basosquamous carcinoma, squamous cell carcinoma of skin, chronic obstructive pulmonary disease, and dyspnoea

The TEAE were considered drug-related in 78.5% of patients (n = 252). TEAE led to treatment discontinuation, dose reduction and interruption in 59 (18.4%), 73 (22.7%) and 98 (30.5%) patients, respectively (Table 2). Serious TEAEs were reported in 87 (27.1%) patients. The serious TEAE were considered drug-related in 4.1% of patients (n = 13) (Table 2). The main reason for treatment interruption and dose reduction was AEs (Table 3).

**Table 3:** Interruptions and dose reductions

	*N* = *321*
Median duration of treatment interruption, month (IQR)	31 (13–91)
Number of patients with at least one therapy interruption, n (%)	156 (48.6)
Number of therapy interruptions, median (range)	1 (1–13)
Reasons for treatment interruptions, n (%)	
AE*	107 (33.3)
Serious AE	3 (0.9)
Patient's wish	30 (9.3)
Tumour progression	7 (2.2)
Unknown	8 (2.5)
Complete response / achieved therapy goal	14 (4.4)
(Un)Availability of care / drug	7 (2.2)
Physician's decision	12 (3.7)
Scheduled interruptions	8 (2.5)
Other	6 (1.9)
Number of patients with at least one dose reduction, n (%)	132 (41.12)
Number of dose reductions, median (range)	1 (1–3)
Reasons for dose reduction, n (%)	
AE^#^	86 (26.8)
Serious AE	1 (0.3)
Patient's wish	12 (3.7)
Tumour progression	2 (0.6)
Unknown	12 (3.7)
Complete response / achieved therapy goal	8 (2.5)
(Un)Availability of care / drug	1 (0.3)
Continuation after interruption	1 (0.3)
Physician's decision	17 (5.3)
Missing	1 (0.3)

^*^TEAEs leading to treatment interruptions that occurred in more than 2% of patients were muscle spasms (*n* = 25, 7.8%), dysgeusia (*n* = 18, 5.6%), nausea (*n* = 13, 4.1%), blood creatine phosphokinase increased (*n* = 10, 3.1%), fatigue (*n* = 8; 2.5%), alopecia (*n* = 8, 2.5%), weight decreased (*n* = 7, 2.2%), decreased appetite (*n* = 7, 2.2%)

^#^TEAEs leading to dose reduction that occurred in more than 2% of patients were muscle spasms (*n* = 29, 9.0%), dysgeusia (*n* = 14, 4.4%), nausea (*n* = 12, 3.7%), alopecia (*n* = 10, 3.1%), fatigue (*n* = 7; 2.2%)

**Fig. 1:**
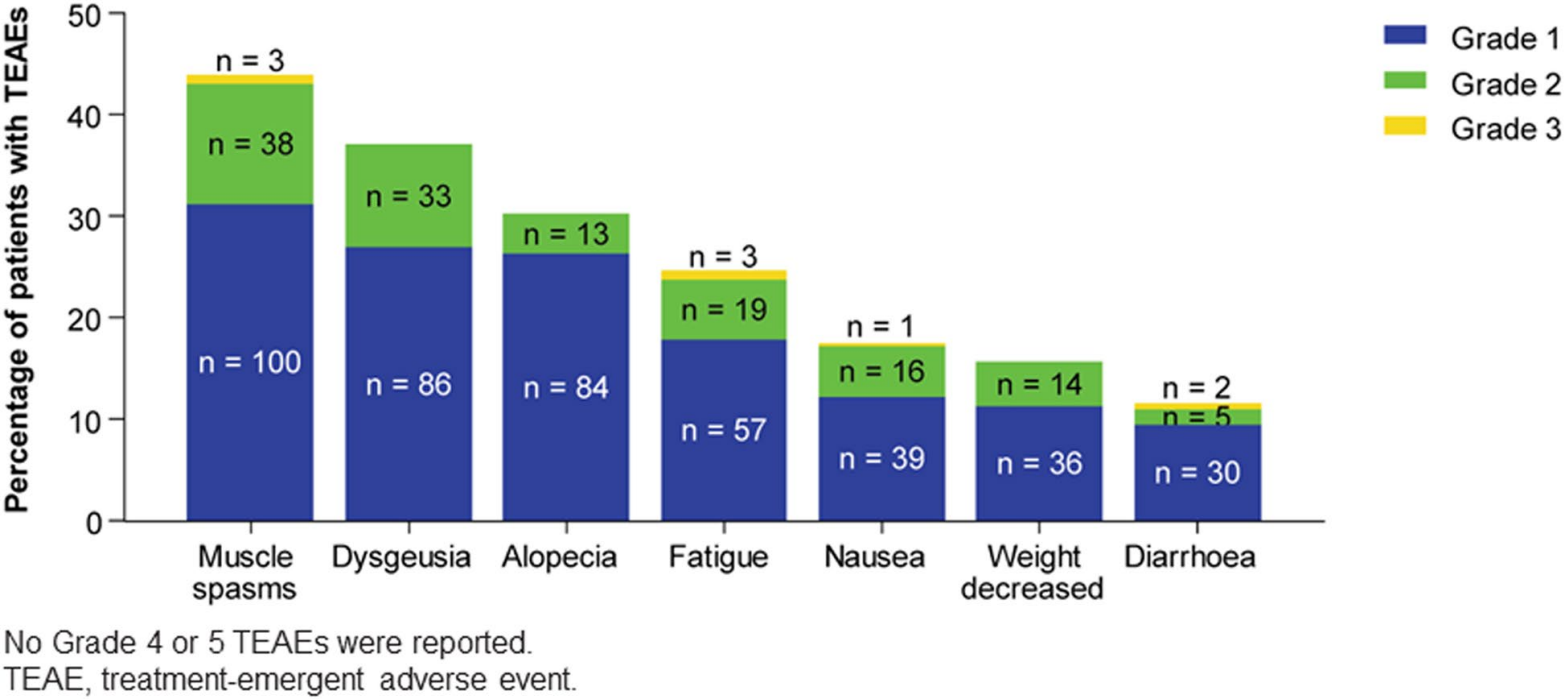
Incidence of common (≥ 10%) TEAEs by severity

**Fig. 2:**
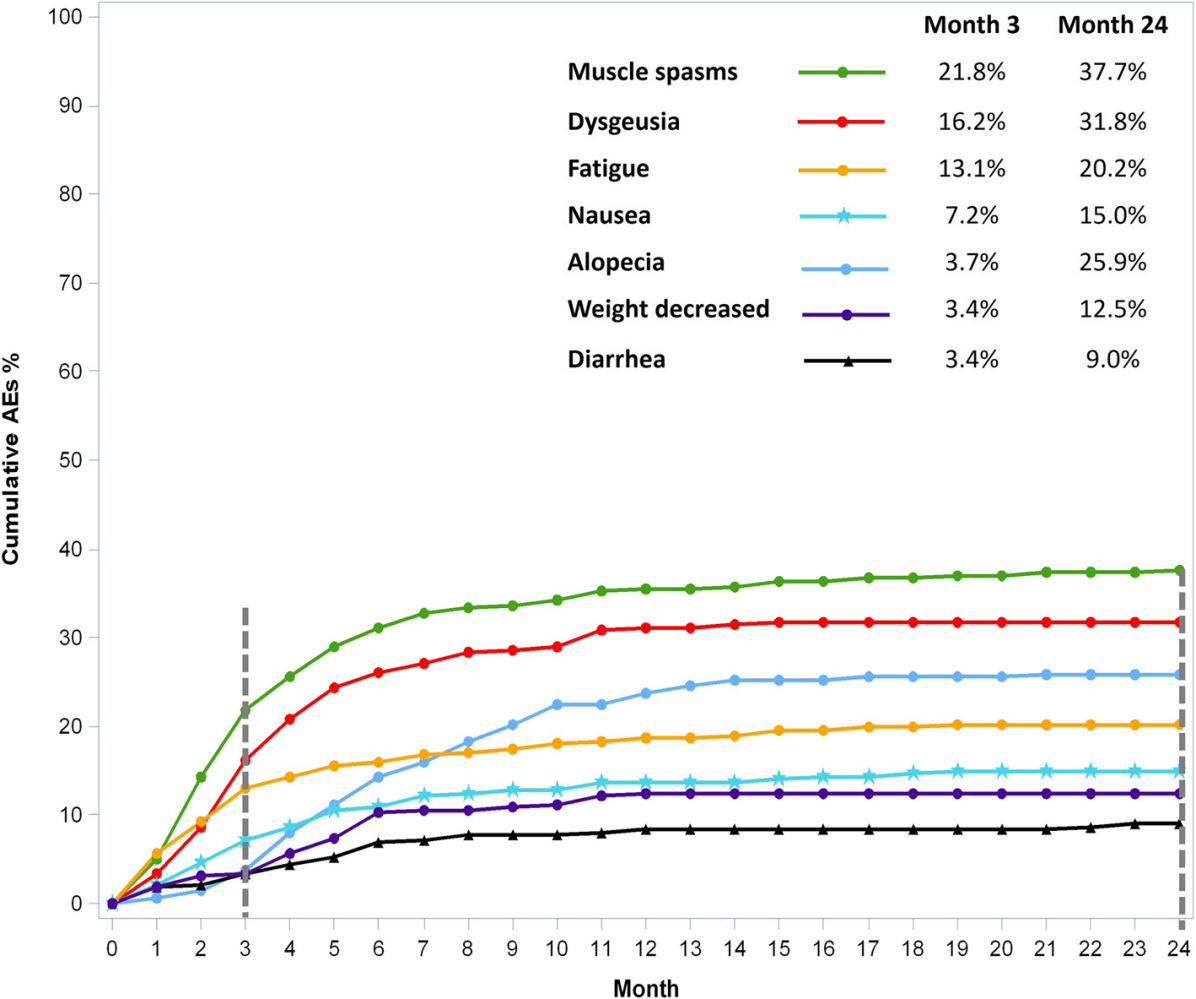
Cumulative onset of TEAEs

Figure 1 summarizes the most common incidences of TEAE, i.e. those occurring in 10% or more of patients, by severity. Most TEAEs recorded were ≤ grade 2. The most common TEAEs were muscle spasms (n = 141; 43.9%), dysgeusia (n = 119; 37.1%), and alopecia (n = 97; 30.2%). The median time to onset of common TEAE was 2.2 months (95% CI: 1.4–3.0) for fatigue, 2.7 months (95% CI: 2.0–3.3) for muscle spasm, 3.0 months (95% CI: 2.5–3.8) for dysgeusia, 3.2 months (95% CI: 2.3–4.6) for nausea, 4.3 months (95% CI: 2.4–5.6) for diarrhoea, 4.4 months (95% CI: 3.7–5.2) for weight decrease and 5.5 months (95% CI: 4.7–6.9) for alopecia. Figure 2 shows the cumulative onset of common TEAE. After 3 months of treatment, the cumulative rates of muscle spasms, dysgeusia, and alopecia were 21.8%, 16.2%, and 3.7%, respectively. The cumulative rate of the most common TEAEs remained approximately stable from month 10 to month 24 of the study. We analysed the worst TEAE outcome among the 284 patients with at least one TEAE at the data cut-off. The worst outcome was “ongoing” for 126 patients, “resolved” for 89 patients, “unknown” for 46 patients, “death” for 17 patients, “recovered with sequelae” for 3 patients and “improved” for 3 patients. Of the 209 patients who ended the sonidegib treatment for a reason other than death or were lost to follow-up, 160 (76.6%) received no further laBCC treatment, while 49 (23.4%) patients underwent surgery (n = 7; 3.3%), radiotherapy (n = 3; 1.4%), other local therapy (n = 4; 1.9%) or systemic therapy (n = 35; 16.7%; of which 26 patients with immunotherapy and 4 with vismodegib) as further laBCC treatment after sonidegib.

## Discussion

This interim analysis of the NISSO post-marketing safety observational study provides valuable information regarding safety and tolerability associated with long-term sonidegib treatment (median follow-up time of 18.9 months).

The safety profile is overall consistent with the safety profile of sonidegib recorded in the BOLT pivotal trial [8]. NISSO has a similar target population to BOLT, except for the proportion of those previously treated with surgery and/or radiotherapy (40% and 10% of the NISSO patients had previously undergone surgery or radiotherapy respectively, compared to 76% and 32% for BOLT) [6]. Following the introduction of HHIs, the target population suitable for their use has indeed experienced a redefinition, which is reflected in clinical practice in the population actually treated with these drugs. Originally, the term ‘locally advanced’ was introduced when patients who were not eligible for surgery and/or radiotherapy were sought for studies with HHIs. The current European BCC guidelines recommend the use of the HHIs sonidegib and vismodegib for patients not amenable to surgery and/or radiotherapy in the EADO stage II (common BCC considered difficult-to-treat for any reasons linked to the patient or tumour and BCCs considered difficult-to-treat because of their number, e.g. Gorlin syndrome) and in the EADO Stage III (large and destructive tumours out of or on critical/functional areas and extremely destructive tumours). Vismodegib is also recommended in EADO Stage IV lesions (mBCC) [5].

Common TEAEs in the NISSO study were muscle spasms, alopecia, dysgeusia, decreased weight, decreased appetite, diarrhoea, fatigue, and nausea. These are expected class effects associated with on-target inhibition of the Hh signalling pathway and are common with other HHIs such as vismodegib [9]. Incidences of the common TEAEs dysgeusia and fatigue were similar to those in the BOLT study, while any other common TEAE occurred in fewer patients [9]. Compared to the vismodegib open-label phase II safety study STEVIE, a lower incidence of muscle spasm, dysgeusia, alopecia, decreased weight and reduced appetite was observed in NISSO, while the rates for fatigue, nausea and diarrhoea were similar [10].

Most TEAEs were of mild or moderate severity and the percentage of patients with serious TEAEs was in line with that reported in the BOLT study [8]. The most common strategies used in clinical practice to improve the tolerability of HHIs and the duration of treatment are dose reductions and interruptions. About 40% and 50% of the NISSO population experienced dose reduction and interruption, respectively. Dose reduction (from the starting daily dose to one capsule every other day), in case this is required to reduce AEs, is only within the label of sonidegib [11]. A retrospective observational study of 82 patients in Spain showed significantly less AEs and comparable clinical effectiveness between daily dose and every other day dose [12], which is consistent with data from the BOLT study [13] and from real life [14]. The percentage of NISSO patients with TEAEs requiring interruption or dose reduction was consistent with the BOLT study, while the proportion of patients with TEAEs leading to discontinuation of sonidegib was lower [8]. The fact that discontinuation rates for HHIs are higher in pivotal studies than in real-world practice is confirmed by the non-interventional NIELS study [15], in which interruptions of treatment until disappearance of the AEs seemed to be the norm. The NIELS study assessed the effectiveness and safety of vismodegib in 66 laBCC patients under real-world conditions in Germany. Permanent discontinuation of treatment due to AEs only occurred in one patient, but 36% of the patients interrupted treatment because of AEs with a median interruption of 7.6 months before re-challenge. This approach of AE management, with interruptions and re-challenge, still led to an objective response rate (ORR) of 74.2% and a median duration of response and median progression-free survival of 15.9 months and 19.1 months, respectively. Two expert consensus papers by Bossi et al. [16] and by Heppt et al. [17] discussed how dose reductions and interruptions followed by re-exposure, together with active AE pharmacological treatment, can be successfully used to manage AEs related to HHI therapy. As HHIs represent, so far, the most effective treatment to achieve an early, high and long-lasting response, the goal is to extend HHI therapy as much as possible [16, 17].

Most NISSO patients experienced the onset of common TEAEs after 3 months of treatment and the cumulative rate remained approximately stable from month 10 to month 24 of the study. At month 3 of sonidegib use, less patients experienced muscle spasms and nausea compared to BOLT. The proportion of patients with other common TEAEs was similar. These NISSO data confirm the existence of a window of opportunity in roughly the first 3 months of treatment in which most patients have not yet experienced the most common AEs but may already have achieved a response. It is indeed known that the median time to response with sonidegib was 1.9 months by investigator review in the BOLT trial [6] and 2.3 months in the retrospective observational PaSoS study [18]. Having a window of time where potentially most patients already achieved response but experienced no or few low-grade AEs nevertheless means that they could have obtained a tumour shrinkage sufficient to make their lesion amenable to local therapies such as surgery or radiotherapy.

Approximately 20% of the NISSO patients (N = 49/209), who discontinued sonidegib treatment for a reason other than death or loss of follow-up, underwent further laBCC treatment, including 7 patients who had surgery and 3 who had radiotherapy. Growing evidence points to the potential use of HHIs as a neoadjuvant approach prior to surgery for laBCC, due to significant tumour shrinkage seen during the pivotal trial [6, 19–22]. Thirty-five of the NISSO patients (16.7%) underwent systemic therapy after sonidegib treatment, mostly cemiplimab. Cemiplimab is the only second-line treatment approved in laBCC and is recommended for patients developing progression while on HHI therapy (resistance) or in case of persisting toxicities despite failure of long-term management of AEs [16, 17]. It is important to note that approximately 80% of the NISSO population did not receive any further laBCC therapy during the follow-up period of the study.

When analysing the reasons for ending sonidegib treatment in the NISSO study, it can be observed that more patients discontinued due to treatment success than to toxicity. This is in line with the results of the French national registry CARADERM that reported sonidegib discontinuation as being more related to clinical benefit rather than AEs [23] and with the high efficacy results from the pivotal trials of HHIs (ORR ranging from 47% for vismodegib to 61% for sonidegib) [24]. As noted by Herms et al., a high discontinuation rate should not be perceived as negative per se as reasons such as satisfactory efficacy or treatment holidays may be significant and cannot be overlooked [25].

About 15% of the NISSO patients had been previously treated with an HHI. Re-challenge with a different HHI, such as switching from vismodegib to sonidegib to improve tolerability, is documented in the literature. In a retrospective single-centre analysis of 36 patients treated with HHIs, Grossmann et al. analysed patients treated with both vismodegib and sonidegib subsequently (and same dosing regimen) showing a reduced occurrence of dysgeusia, alopecia, muscle spasms, weight loss and fatigue during treatment with sonidegib [26]. Additionally, multiple case reports support switching to sonidegib to achieve a better safety profile [27–31].

A *post-hoc* analysis of the sonidegib BOLT study and the expanded-access, open-label vismodegib study revealed that patients treated with sonidegib had a later median time to onset for all common AEs than patients treated with vismodegib, except fatigue and weight decrease [9]. After 3 treatment cycles of vismodegib, the cumulative rates of the most common AEs muscle spasm, dysgeusia, and alopecia were approximately 60%, 60%, and 25%, respectively, while these rates for sonidegib were 32.9%, 15.2%, and 5.1%, respectively. Assessment of published data from pivotal studies of sonidegib and vismodegib showed that sonidegib had slightly less frequent and less severe common AEs compared with vismodegib at final analyses [9, 24].

While the results of the interim analysis of the NISSO study support previous findings and fill data gaps, it is important to take into consideration that there are limitations of the presented data mainly based on the nature of an observational study. The most important aspects to note are the lack of a comparator arm and the lack of an independent central review.

## Conclusions

The interim results of the NISSO observational study provides real-world evidence of the safety profile of sonidegib in the widest patient population so far, showing that the tolerability of sonidegib is manageable in routine clinical practice consistent with that previously reported.

## Data Availability

The data that support the findings of this study are available from Sun Pharmaceuticals Industries Limited, but restrictions apply to the availability of these data and so are not publicly available. Data are however available from the authors upon reasonable request and with permission of Sun Pharmaceuticals Industries Limited.

## Abbreviations

AE: Adverse event
BCC: Basal cell carcinoma
CI: Confidence interval
CR: Complete response
EADO: European Association of Dermato-Oncology
EMA: European Medicines Agency
FDA: US Food and Drug Administration
Hh: Hedgehog
HHI: Hedgehog inhibitor
IQR: Interquartile range
laBCC: Locally advanced BCC
mBCC: Metastatic BCC
NBCCS: Nevoid basal cell carcinoma syndrome
NMSC: Non-melanoma skin cancer
ORR: Objective response rate
PASS: Post-authorization safety study
PD-1: Programmed cell death 1
PR: Partial response
PTCH: Patched
TEAE: Treatment-emergent adverse event
